# Chronic heat stress induces the expression of *HSP* genes in the retina of chickens (*Gallus gallus*)

**DOI:** 10.3389/fgene.2023.1085590

**Published:** 2023-04-03

**Authors:** Nasmah K. Bastaki, Taybha A. Albarjas, Fatma A. Almoosa, Amani M. Al-Adsani

**Affiliations:** Department of Biological Sciences, Faculty of Science, Kuwait University, Kuwait City, Kuwait

**Keywords:** chronic heat stress, HSP genes, chicken, retina, RT-qPCR

## Abstract

**Introduction:** Chronic heat stress during summer is a major challenge imposed by global warming. Chickens are more sensitive to heat stress than mammals because they lack sweat glands. Thus, chickens are more susceptible to heat stress during summer than other seasons. Induction of heat shock protein (HSP) genes is one of the primary defense mechanisms against heat stress. Tissue-specific responses exhibited by different classes of HSPs upon exposure to heat stress have been reported previously in different tissues including the heart, kidney, intestine, blood, and muscle, but not in the retina. Therefore, this study aimed to investigate the expression levels of *HSP27*, *HSP40*, *HSP60*, *HSP70*, and *HSP90* in the retina under chronic heat stress.

**Methods:** This study was conducted during the summers of 2020 and 2021 in Kuwait. Chickens (*Gallus gallus*) were divided into control and heat-treated groups and sacrificed at different developmental stages. Retinas were extracted and analyzed by using Real Time quantitative Polymerase Chain Reaction (RT-qPCR).

**Results:** Our results from the summer of 2021 were similar to that from the summer of 2020, regardless of whether *GAPDH* or *RPL5* was used as a gene normalizer. All five HSP genes were upregulated in the retina of 21-day-old heat-treated chickens and stayed upregulated until 35 days of age, with the exception of *HSP40*, which was downregulated. The addition of two more developmental stages in the summer of 2021 showed that at 14 days, all HSP genes were upregulated in the retina of heat-treated chickens. In contrast, at 28 days, *HSP27* and *HSP40* were downregulated, whereas *HSP60*, *HSP70*, and *HSP90* were upregulated. Furthermore, our results showed that under chronic heat stress, the highest upregulation of HSP genes was seen at the earliest developmental stages.

**Discussion:** To the best of our knowledge, this is the first study to report the expression levels of *HSP27*, *HSP40*, *HSP60*, *HSP70*, and *HSP90* in the retina under chronic heat stress. Some of our results match the previously reported expression levels of some HSPs in other tissues under heat stress. These results suggest that HSP gene expression can be used as a biomarker for chronic heat stress in the retina.

## 1 Introduction

Increases in global temperature pose severe environmental challenges. Heat stress is one of the most challenging environmental stresses imposed by global warming and the rise in global temperature. Heat stress occurs in two ways: 1) acute heat stress, which is characterized by a brief period of intense environmental temperatures and 2) chronic heat stress, which is characterized by prolonged high temperatures. Heat stress affects domestic animal production, especially in commercial poultry, causing massive economic losses (Temim et al., 2000; Das et al., 2016; Shehata et al., 2020; Nawaz et al., 2021).

Kuwait is a small country in the Arabian Gulf region known for its dry and hot desert climate with very high temperatures during summers. Kuwait’s extremely hot weather affects the desert and marine ecosystems, with daily maximum temperatures exceeding their long-term averages for several consecutive days (Nasrallah et al., 2004; Al-Awadhi et al., 2005). Summer in Kuwait lasts from the end of May until mid-September, with the hottest months being June–August (Sedaghat et al., 2021). Hence, chronic heat stress can be easily induced in Kuwait during the summer. In 2016, Asia’s highest temperature was recorded in Kuwait at 54.0°C (Alahmad et al., 2020). In 2021, Kuwait City recorded 53.2°C, the world’s highest recorded temperature in the summer of 2021. (https://gulfnews.com/world/gulf/kuwait/kuwait-registers-highest-temperature-on-earth-for-2021-1.80222855).

Chickens (*Gallus gallus)* are homoeothermic animals with a maintenance body temperature in the range of 41–42°C. Chickens lack sweat glands and are therefore more sensitive to high temperatures and heat stress than other animals (Murugesan et al., 2017; Cândido et al., 2020). During heat stress, metabolic heat increases, triggering hyperthermia (Nawaz et al., 2021), which is a combined effect of high environmental temperature and humidity (Olcina et al., 2019). Hyperthermic chickens are characterized by poor growth owing to damaged skeletal muscle cells and impaired protein synthesis. Additionally, they are immunocompromised and therefore, may suffer from fatal diseases (Shehata et al., 2020; Nawaz et al., 2021).

One of the primary defense mechanisms against hyperthermia is the activation of heat shock proteins *(*HSPs), also called “stress proteins”. HSPs are the major players in adaptive responses to heat stress and are considered the first line of protection for cells exposed to heat stress (Piri et al., 2016; Cedraz et al., 2017; Murugesan et al., 2017; Dubrez et al., 2020; Siddiqui et al., 2020a; Siddiqui et al., 2020b). HSPs are a large group of molecular chaperones that can block signaling cascades that affect cell death, protect essential signaling pathways needed for cell survival, or inhibit misfolded protein formation and aggregation. HSPs are classified into five families based on their molecular weight: HSP27, HSP60, HSP70, HSP90, and HSP110 (Bukau et al., 2000; Oshlag et al., 2013; Dubrez et al., 2020).

Previous studies on the effects of heat stress on tissue-specific responses in chickens have shown that HSP expression is induced upon exposure to acute and chronic heat stress. The expression levels of *HSP70*, *HSP60,* and *HSP47* were significantly high in different sections of the small intestine of chickens (duodenum, ileum, and jejunum) upon exposure to acute heat stress (Siddiqui et al., 2020). Moreover, there were differences in the gene expression of HSPs in different regions of the chicken intestine. For example, the mRNA and protein expression of *HSP70* and *HSP90* increased in the ileum more than that in the jejunum upon exposure to heat stress. A study confirmed that the gastrointestinal tract of chickens is primarily responsive to heat stress (Varasteh et al., 2015). In another report, *HSP70* and *HSP90* genes were highly expressed in the muscle of chronic heat-stressed chickens when compared to the muscle of control chickens (Cedraz et al., 2017). Tissue-specific responses to acute and chronic heat stress have also been documented in the hearts and livers of chickens. For example, during chronic heat treatment, HSP70 mRNA levels increased in the heart, but HSP90 mRNA levels decreased. During acute heat challenge, the expression of *HSP70* was highly increased in the liver, and *HSP90* expression is increased by chronic heat treatment (Xie et al., 2014).

The retina is a sensory nerve membrane that lines the back of the eye to facilitate a connection between nerve impulses and the brain *via* the optic nerve. The chicken retina comprises neuronal cells that are precisely organized within defined layers. These neuronal cells and layers are conserved across all vertebrates (Yamagata et al., 2021). There are numerous studies on HSPs in retinal responses to eye diseases or other environmental stressors, and most of these studies have reported that HSPs protect the retina from damage. For example, HSPs have been shown to play a protective role against apoptosis in the retinas of diabetic retinopathy (Mohammad and Kowluru, 2011; Pinach et al., 2013; Reddy et al., 2013; Jo et al., 2014). Under hypoxic stress, HSP27 is upregulated in the retina and serves as a cytoprotective factor for preventing retinal ischemic damage (Whitlock et al., 2005). Moreover, upon exposure to bright light, HSP27 is shown to protect the photoreceptors by decreasing apoptosis of retinal cells (Chien et al., 2017). HSP70 also plays a neuroprotective role in retinal ganglion cells in hyperthermia, glaucoma, and hypertension (Piri et al., 2016).

Research on HSPs in the retinas of chickens exposed to heat stress is limited, but a few studies have demonstrated their roles in other organisms. For example, in one study, HSP70 mRNA and proteins were localized to the photoreceptor layer of the rat retina after brief exposure to whole-body hyperthermic treatment (Tytell et al., 1994). In another study, HSP70 mRNA levels increased in the retina of zebrafish after 30 min of heat shock induction at 37°C (Fujikawa et al., 2012). However, further studies are needed to explore the effects of HSPs in the retina in response to heat stress.

The objective of the present study was to determine the effect of chronic heat stress on the retina of chickens during summer season in Kuwait. This study was conducted over 2 years, during the summers of 2020 (August–September) and 2021(June–July), to assess the effect(s) of retinal expression of *HSP27*, *HSP40*, *HSP60*, *HSP70*, and *HSP90* in chickens.

## 2 Materials and methods

### 2.1 Ethical approval

The chickens used in the present study were under the care and supervision of a poultry veterinarian at Kuwait University. The chickens were handled, maintained, and experimented with according to the “Instructions Guide” for the Care and Use of Laboratory Animals (National Research Council, 1996). The experimental procedures were approved by the Ethics Committee on Animal Use at Kuwait University and ECULA Ethical Committee for the Use of Laboratory Animals (DBS/IRB(ECULA)20-005).

### 2.2 Animals

**FIGURE 1:**
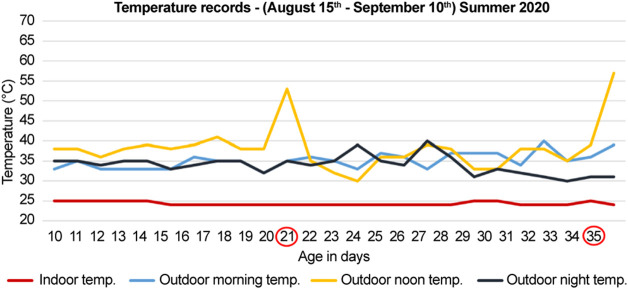
Temperature records for summer of 2020 (months of August and September). The documented temperature during the morning, noon, and night for the control (indoor) and heat-treated (outdoor) groups. The first group was sacrificed at 21-days, and the second group was sacrificed at 35-days (circled in red). The peaks indicate direct exposure to sun at noon for 10 min before sacrifice of the heat-treated chickens.

**FIGURE 2:**
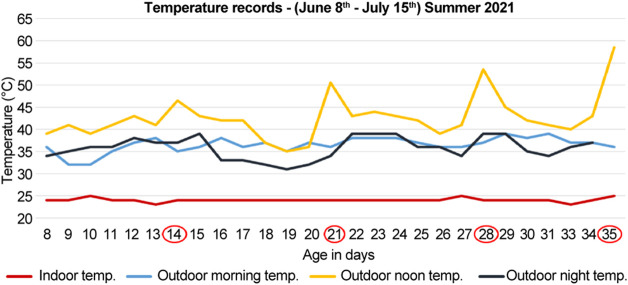
Temperature records for the summer of 2021 (months of June and July). The documented temperature during the morning, noon, and night for the control (indoor) and heat-treated (outdoor) groups. The first, second, third, and fourth groups were sacrificed at 14-, 21-, 28-, and 35-days (circled in red), respectively. The peaks indicate direct exposure to sun at noon for 10 min before sacrifice of the heat-treated chickens.

In the present study, 68 local Kuwaiti chicks (*Gallus gallus domesticus*) were used across experiments conducted in the summers of 2020 and 2021. Newly hatched chicks (8–10 days old) were purchased from local farms in Kuwait and divided into two groups: the control (indoor) group, in which the temperature was maintained at 25°C, and the heat-treated (outdoor) group, in which the temperature fluctuated according to the outside temperature during summer in Kuwait (35–50°C). Temperatures were recorded thrice every day (morning, afternoon, and evening) (Figures 1, 2). The chicks were carefully observed for behavioral changes, especially in the outdoor group, to assess normal behavior (such as eating, breathing, and moving) and avoid sudden deaths caused by excessive heat. The chicks in both groups had full access to food and water at all times.

### 2.3 Distribution, number of chicks, and treatment end point

#### 2.3.1 Summer of 2020

During the summer of 2020 (months of August and September), 28 chicks were procured and divided into two groups; each group had seven control (indoor) and seven heat-treated (outdoor) chicks. The first and second groups were sacrificed at 21- and 35-days, respectively (Figure 1). The number of deaths were recorded. The recorded mortality in each group ranged between 2–4 chicks. Therefore, three chicks were used in each group to standardize the number of chicks in each group.

The outdoor noon temperatures ranged from to 36–41°C, and for the indoor group, it was maintained at 25°C. Before sacrifice, the outdoor chickens were further exposed to the direct sun for 10 min at a temperature of 53°C in the 21-days old group and 57°C in the 35-days old group (Figure 1). The indoor groups were sacrificed indoors at 25°C at the same time (Figure 1).

#### 2.3.2 Summer of 2021

During the summer of 2021 (months of June and July), 40 chicks were divided into four groups; each group had five control (indoor) and five heat-treated (outdoor) chicks. The first, second, third, and fourth groups were sacrificed at 14-, 21-, 28-, and 35-days, respectively (Figure 2). The number of deaths were recorded. The recorded mortality in each group ranged from 1–2 chicks. Therefore, three chicks were used in each group to standardize the number of chicks in each group.

The noon temperatures ranged from 39 to 45°C for the heat-treated group, and 25°C for the control group. Before sacrifice, the heat-treated chickens were further exposed to direct sun for 10 min at a temperature of 46.5°C for the 14-days old, 50.5°C for the 21-days old, 53.5°C for the 28-days old, and 58.5°C for 35-days old chicks (Figure 2). The control groups were sacrificed indoors at 25°C at the same time (Figure 2).

Chickens were sacrificed by cervical dislocation, their eyes were collected, and their retinas were excised and immediately processed for RNA extraction.

### 2.4 RNA extraction and cDNA synthesis

RNA was extracted using TRIzol reagent according to the manufacturer’s protocol (15596018, Invitrogen, Waltham, MA, United States) and then resuspended in 50 µL diethylpyrocarbonate-treated water. The quantity and quality of the RNA samples were checked using a Nanodrop 8,000 spectrophotometer (ND-8000-GL, Thermo Fisher Scientific, Inc., Waltham, MA, United States). Genomic DNA was removed using a TURBO DNA-free kit (AM 1907, Invitrogen). A cDNA reverse transcription kit was used to synthesize cDNA and oligo dT primers, according to the manufacturer’s protocol (4368814, Applied Biosystems, Waltham, MA, United States). The quantity and quality of cDNA samples were evaluated using a Nanodrop 8,000 spectrophotometer.

### 2.5 Reverse transcriptase PCR (RT-PCR)

Forward and reverse primers for the reference and HSP genes were designed using the primer design tool at NCBI (http://www.ncbi.nlm.nih.gov/tools/primer-blast/) and used to amplify approximately 150–200 bp products (Table 1). For polymerase chain reaction (PCR), Platinum™ Green Hot Start PCR 2X master mix (13001014, Invitrogen) was used according to the manufacturer’s protocol. The cDNA concentration for all developmental stages was maintained at 25 ng for all PCR reactions. The PCR conditions were as follows: initial denaturation at 94°C for 2 min; 35 cycles of denaturation at 94°C for 30 s, annealing at 55°C for 30 s, and extension at 72°C for 30 s; and final extension at 72°C for 5 min. For gel electrophoresis, 12 µL of each PCR reaction was loaded onto a 1.5% agarose gel and stained with SYBR safe DNA stain (S33102, Invitrogen).

**TABLE 1:** Description of all primers related to *Gallus gallus* HSP genes and the reference genes used for RT-qPCR analysis.

Gene	Full name	Sequence (5′–3′)	Function
*HSP27*	Heat Shock Protein 27	F: ACA​CGA​GGA​GAA​ACA​GGA​TGA​G	Heat Shock Response
		R: ACTGGATGGCTGGCTTGG	
*HSP40*	Heat Shock Protein 40	F: GGCATTCAACAGCATAGA	Heat Shock Response
		R: TTC​ACA​TCC​CCA​AGT​TTA​GG	
*HSP60*	Heat Shock Protein 60	F: TTG​ATG​GAG​AAG​CCC​TCA​GC	Heat Shock Response
		R: TCC​TCT​CCA​AAC​ACA​GCA​CC	
*HSP70*	Heat Shock Protein 70	F: AAG​GGT​AAG​CAC​AAG​CGT​GA	Heat Shock Response
		R: GCA​CGA​GTG​ATG​GAG​GTG​TA	
*HSP90*	Heat Shock Protein 90	F: TCC​TGT​CCT​GGC​TTT​AGT​TT	Heat Shock Response
		R: AGGTGGCATCTCCTCGGT	
*GAPDH*	Glyceraldehyde-3 Phosphate Dehydrogenase	F: AGAACATCATCCCAGCGT	Reference Gene
		R: AGC​CTT​CAC​TAC​CCT​CTT​G	
*HPRT1*	Hypoxanthine Phospho-Ribosyl Transferase 1	F: AGC​CCC​ATC​GTC​ATA​TGC​T	Reference Gene
		R: AGC​CCC​ATC​GTC​ATA​TGC​T	
*BMP2*	Bone Morphogenetic Protein 2	F: GCC​AGA​AAC​AAG​TGG​GAA​AA	Reference Gene
		R: TAC​GGT​GAT​GGT​AGC​TGC​TG	
*B-Actin*	Beta-Actin	F: ACCCCAAAGCCAACAGA	Reference Gene
		R: CCA​GAG​TCC​ATC​ACA​ATA​CC	
*RPL5*	Ribosomal Protein L5	F: AAT​ATA​ACG​CCT​GAT​GGG​ATG​G	Reference Gene
		R: CTT​GAC​TTC​TCT​CTT​GGG​TTT​CT	

Abbreviations: F, forward; R, reverse.

### 2.6 Real-time quantitative PCR (RT-qPCR)

RT-qPCR was performed using a Bio-Rad CFX96 Real-Time system (C1000 Touch Thermal Cycle, Bio-Rad, Singapore). Reactions were performed in a 10 µL reaction mixture containing 5 µL PowerUp SYBR Green Master-Mix 2X (A25779, Applied Biosystems), 2 µL of 10 ng cDNA, 1 µL of each specific primer (10 µM), and 1 µL of molecular-grade water. Gene amplification was performed in duplicates, and each PCR run was performed thrice. A negative control (ultrapure water) was used in each assay. The PCR conditions were as follows: 50°C for 2 min; 40 cycles of 95°C for 2 min, 95°C for 15 s, 57°C for 15 s, 72°C for 1 min; and an extra step of melting analysis for each sample at 65°C for 5 s and 95°C for 5 s.

### 2.7 Statistical analysis of target genes

The Ct value was computed using Bio-Rad CFX96 software to quantify mRNA for each gene, and the average Ct values were calculated for each sample group. The mRNA expression as the ΔCt value (Ct target gene − Ct reference gene) was calculated for each HSP gene. Subsequently, the normalized relative expression ∆∆Ct (∆Ct heat-treated sample − ∆Ct control sample) was calculated for each gene. The fold change 2^−ΔΔCT^ was then obtained for each HSP gene. Finally, statistical analysis was performed by using Student’s t-test with GraphPad Prism version 9 (GraphPad Software Inc., San Diego, CA, United States) to obtain the *p*-value and standard deviation for expression of each HSP gene. Statistical significance was set at *p* < 0.05.

## 3 Results

### 3.1 Specificity and efficiency of the reference genes and the choice of a normalizer for the RT-qPCR

**FIGURE 3:**
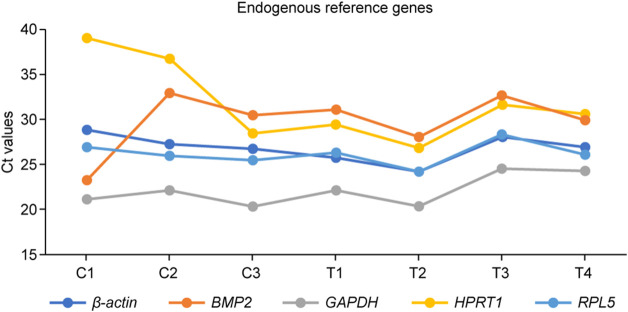
The threshold cycle (Ct) values for the five endogenous reference genes tested before use in the summer of 2021. C1–C3: retina collected from control (indoor) chicks, T1–T4: retina collected from heat-treated (outdoor) chicks.

**FIGURE 4:**
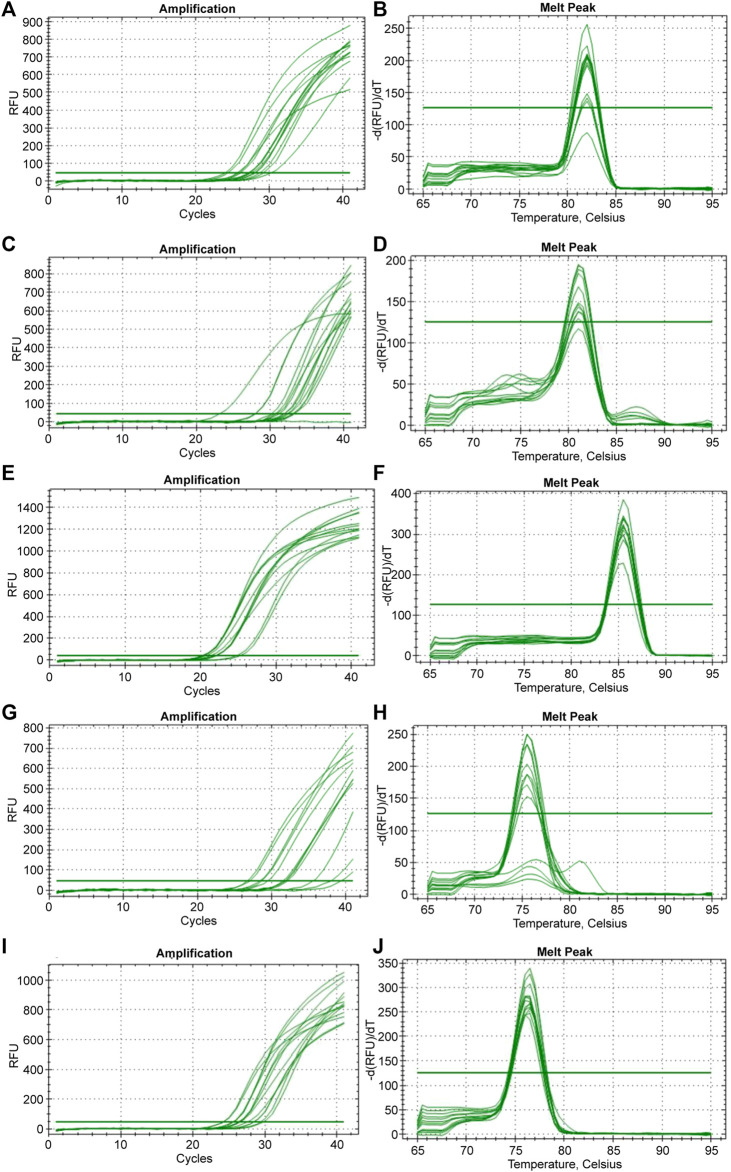
The amplification and dissociation of the efficiency test (melt peak) for the five reference genes tested in the study: **(A, B)** *B-actin*, **(C, D)** *BMP2*, **(E, F)** *GAPDH*, **(G, H)** *HPRT1*, and **(I, J)** *RPL5*.

Five reference genes (*B-actin*, *BMP2*, *GAPDH*, *HPRT1*, and *RPL5*) were tested for their real-time amplification patterns in the control and heat-treated chickens. We found that *GAPDH* had the lowest and most consistent cycle threshold (Ct) values in the control and treated groups, followed by *RPL5* and *B-actin* (Figure 3). *BMP2* and *HPRT1* had very high and variable Ct values in both the control and treated groups. The dissociation curves for *GAPDH* (Figure 4F), *RPL5* (Figure 4J), and *B-actin* (Figure 4B) showed one peak, indicating primer specificity for the target genes. However, the dissociation curves for *BMP2* (Figure 4D) and *HPRT1* (Figure 4H) showed small peaks in addition to the main peak, indicating poor specificity of the primers for the target genes. The amplification curves for the five genes (Figures 4A, C, E, G, I) confirm that *GAPDH*, followed by *RPL5* and then *B-actin*, had the best amplification patterns with consistent Ct values.

Based on these results, *GAPDH* was used as a reference gene normalizer for all RT-qPCR-based gene expression analyses in the summer of 2020. *RPL5* was chosen as a reference gene normalizer for all RT-qPCR-based gene expression analyses in the summer of 2021.

### 3.2 Relative expression of HSP genes in the summer of 2020

**FIGURE 5:**
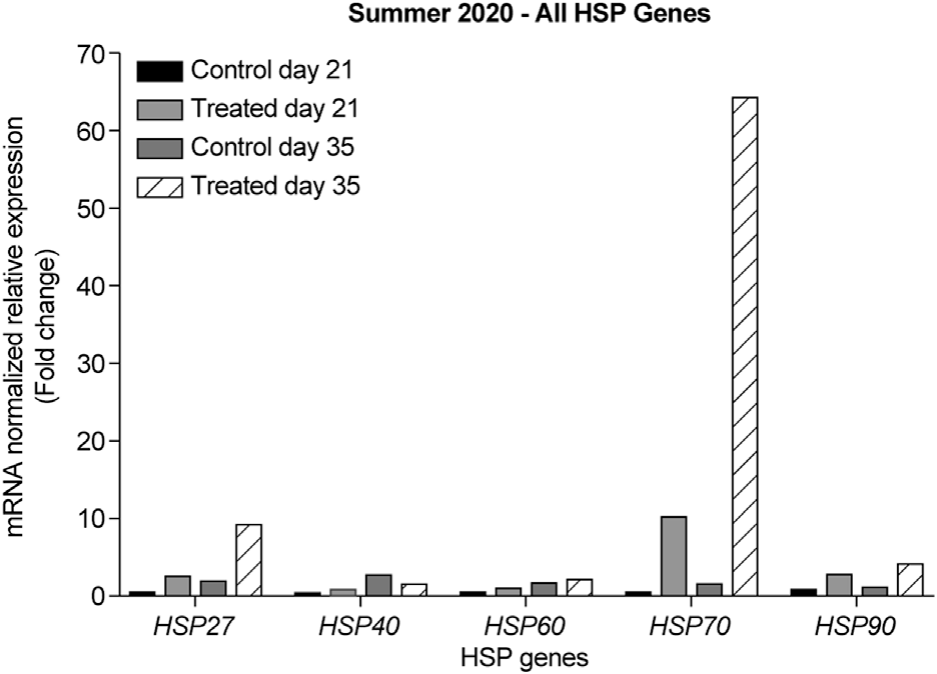
ΔΔ Ct of the relative normalized expression of *HSP27*, *HSP40*, *HSP60*, *HSP70*, and *HSP90* against *GAPDH* in the control (indoor) vs. heat-treated (outdoor) chicks in the summer of 2020, using retinas from 21- and 35-days old chicks. (*n* = 3 in each group). HSP, heat shock protein.

In the summer of 2020, at 21-days, all HSP genes were upregulated in the retinas of the heat-treated group when compared to that of the control group (Figure 5, light grey bars). The highest upregulation was observed for *HSP70*. At 35-days, all HSP genes were upregulated, except for *HSP40*, which was downregulated (Figure 5, gray and black striped bars). The highest upregulation was observed for *HSP70*.

### 3.3 Relative expression of HSP genes in the summer of 2021

**FIGURE 6:**
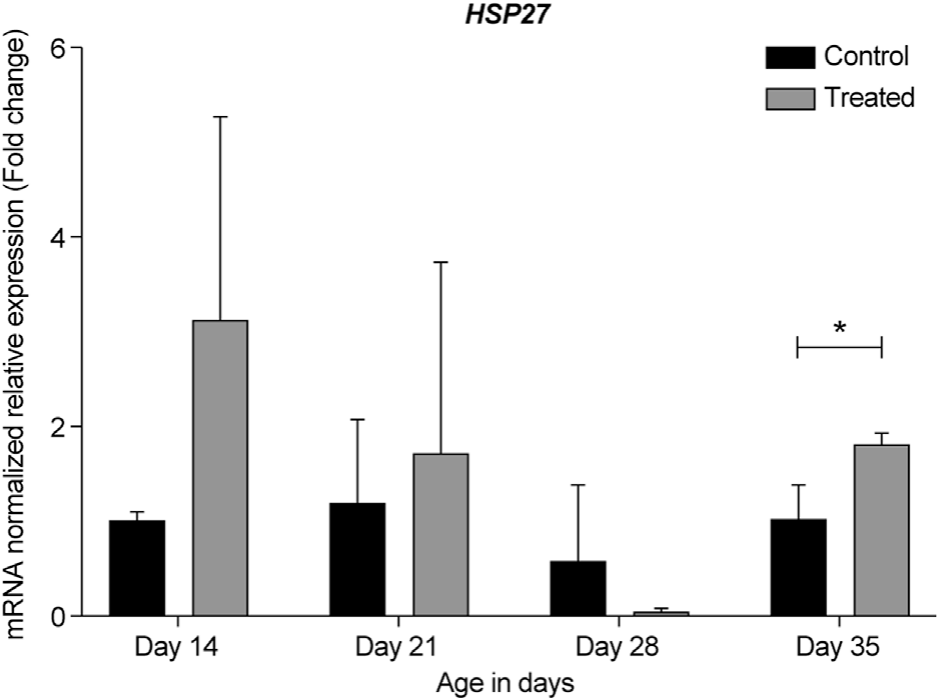
ΔΔ Ct of the relative normalized expression for *HSP27* in the control (indoor) group and heat-treated (outdoor) chickens at 14-, 21-, 28-, and 35-days of age in the summer of 2021 (*n* = 3 in each group, *p < 0.05). HSP, heat shock protein.

**FIGURE 7:**
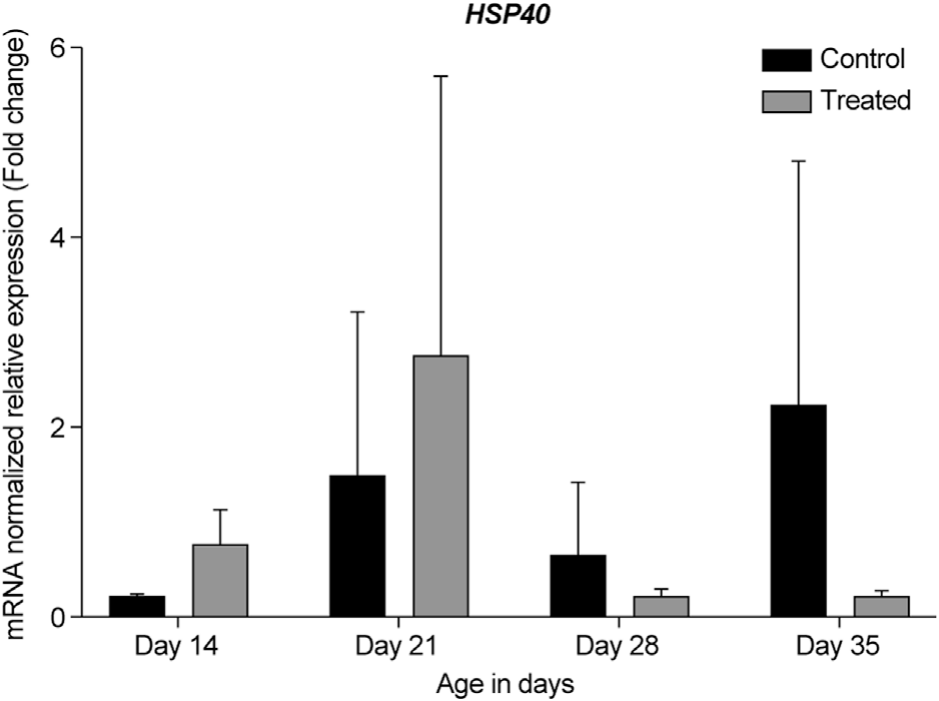
ΔΔ Ct of the relative normalized expression for *HSP40* in the control (indoor) group and heat-treated (outdoor) chickens at 14-, 21-, 28-, and 35-days of age in the summer of 2021. (*n* = 3 in each group). HSP, heat shock protein.

**FIGURE 8:**
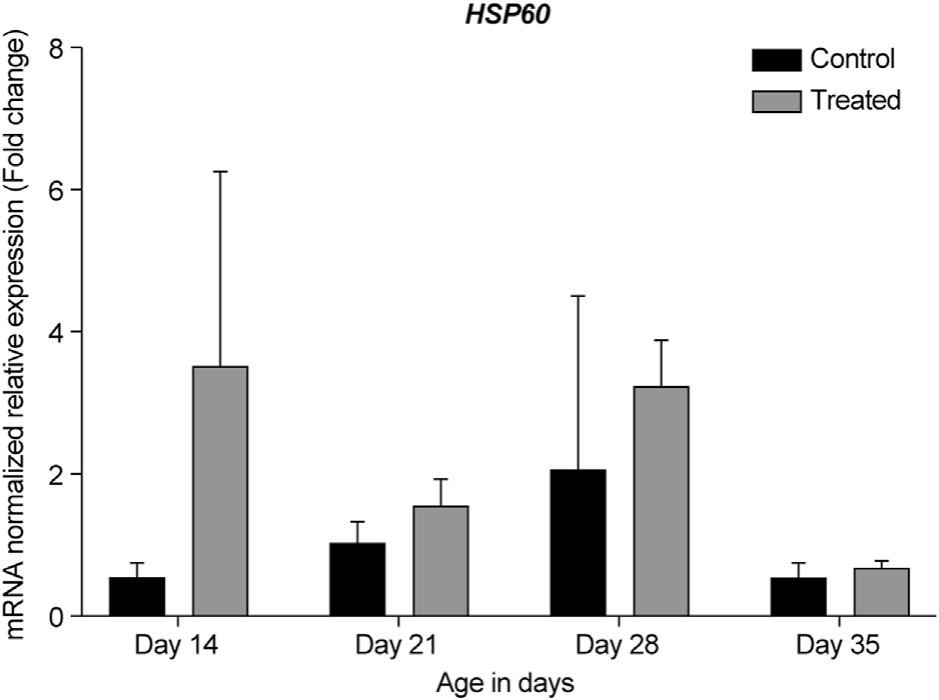
ΔΔ Ct of the relative normalized expression for *HSP60* in the control (indoor) group and heat-treated (outdoor) chickens at 14-, 21-, 28-, and 35-days of age in the summer of 2021 (*n* = 3 in each group). HSP, heat shock protein.

**FIGURE 9:**
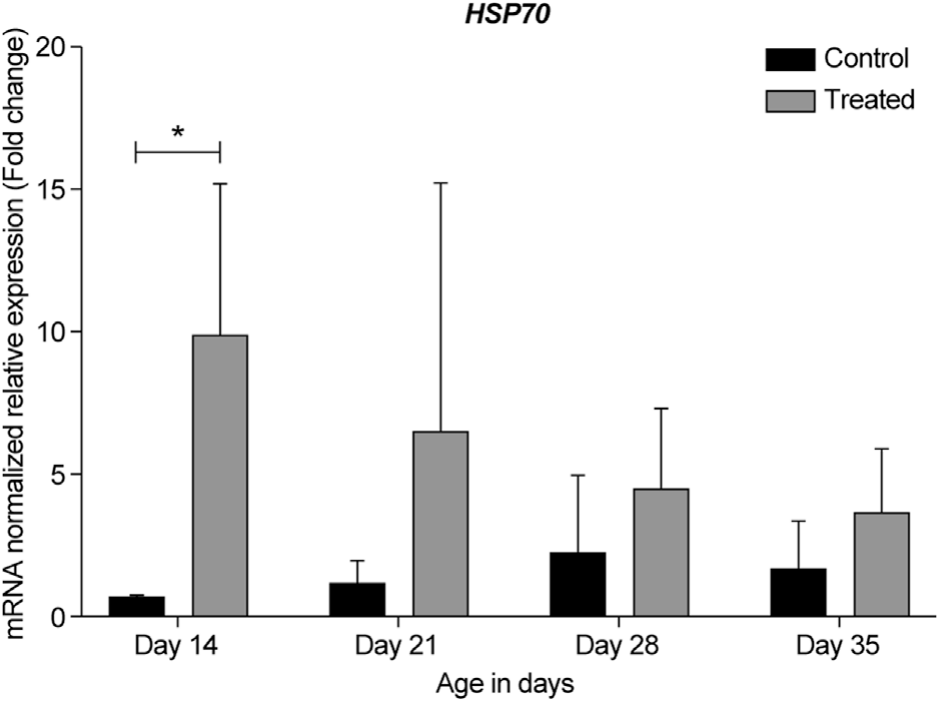
ΔΔ Ct of the relative normalized expression for *HSP70* in the control (indoor) group and heat-treated (outdoor) chickens at 14-, 21-, 28-, and 35-days of age in the summer of 2021 (*n* = 3 in each group, *p < 0.05). HSP, heat shock protein.

**FIGURE 10:**
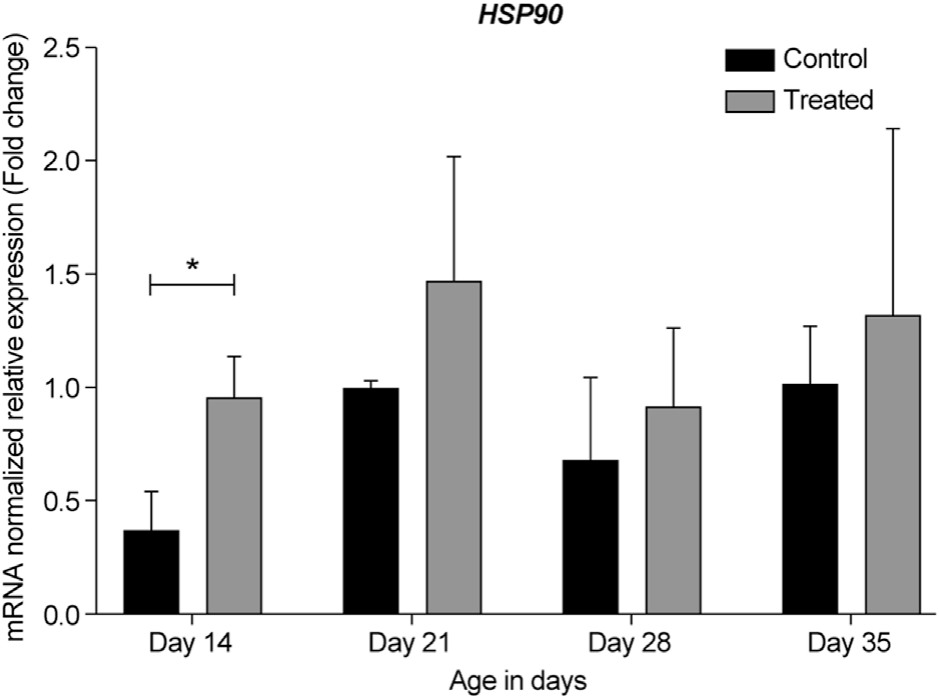
ΔΔ Ct of the relative normalized expression for *HSP90* in the control (indoor) group and heat-treated (outdoor) chickens at 14-, 21-, 28-, and 35-days of age in the summer of 2021 (*n* = 3 in each group, *p < 0.05). HSP, heat shock protein.

In the summer of 2021, at 14- and 21-days, all HSP genes were upregulated in the retinas of the heat-treated group when compared to that of the control group (Figures 6–10, Days 14 and 21). At 28 days, *HSP27* and *HSP40* were downregulated in the retinas of the heat-treated group compared to that of the control group. In contrast, at 28 days, the expression levels of *HSP60*, *HSP70*, and *HSP90* were upregulated in the retinas of the heat-treated group when compared to that of the control group (Figures 6–10, Day 28). At 35 days, all HSP genes were upregulated, except for *HSP40*, which was downregulated (Figures 6–10, Day 35).

The expression profile for HSP genes at 21- and 35-days in the summers of 2020 and 2021 was similar, regardless of the reference gene normalizer, which was *GAPDH* in the summer of 2020 and *RPL5* in the summer of 2021. Moreover, a considerable upregulation of *HSP27*, *HSP60*, and *HSP70* was observed in the earliest developmental stage, i.e., 14-day-old chicks (Figures 6, 8, 9, respectively).

## 4 Discussion

Extreme hot weather during summers is becoming a concern worldwide; the past 7 years have recorded the highest temperatures globally, and it is expected that upcoming years will continue to record high temperatures (Edmondson et al., 2022). The present study was conducted in Kuwait, a desert country with a hyper-arid climate, where temperatures frequently rise above 50°C during summer (Alahmad et al., 2020).

Extreme hot weather can induce chronic heat stress in all organisms. Heat stress triggers various adaptive physiological and cellular mechanisms, primarily the induction of HSPs to prevent hyperthermia, cellular damage, and death (Bouchama et al., 2017; Murugesan et al., 2017). Previous studies on the effects of heat stress on tissue-specific responses in chickens have demonstrated that exposure to heat stress severely affects different organs and tissues. The expression of HSPs is induced in various organs and tissues upon exposure to acute and chronic heat stress, such as the kidneys (Sikand et al., 2012), intestine (Varasteh et al., 2015; Siddiqui et al., 2020), muscle (Cedraz et al., 2017), heart, and liver (Xie et al., 2014). However, studies on the role of HSPs in the retinal response to heat stress are limited. Therefore, this study intended to determine the effect of chronic heat stress on the expression of HSP genes in the retina of chickens during the summer season.

In the summer of 2020, during the months of August and September, the morning, noon, and night temperatures ranged between, 33–39°C, 36–41°C, and 31–39°C, respectively (Figure 1). Likewise, in the summer of 2021, during the months of June and July, the temperatures ranged between 32 and 39°C in the morning, 39–45°C at noon, and 32–39°C in the evening (Figure 2). Therefore, the temperatures were similar in both years regardless of the different summer months in our study, except for the noon temperatures in 2021 summer, which were higher by approximately 3°–4°C than that of 2020 summer. Moreover, the mortality was unaffected by chronic heat stress in both years, as the number of recorded deaths was equal in both control groups and heat-treated groups in 2020 and 2021.

Before RT-qPCR analysis of retinal HSPs, the best reference gene as a normalizer for the experiment was tested and selected accordingly. *GAPDH* and *B-actin* are routinely used to normalize RT-qPCR data (Sikand et al., 2012). However, studies have suggested that the best reference gene may vary depending on the experimental conditions and tissue types used. Therefore, the reference gene selected should be suitable for the investigation of the tissue, and the study design (Dheda et al., 2004; Caracausi et al., 2017; Gromboni et al., 2020). Five endogenous reference genes were tested in this study: *GAPDH* (glyceraldehyde 3-phosphate dehydrogenase), *B-actin* (beta-actin)*, RPL5* (ribosomal protein L5), *BMP2* (bone morphogenetic protein 2), and *HPRT1* (hypoxanthine Phospho-Ribosyl transferase 1). A study on retinas from rats exposed to cyclic hyperoxia reported that *HPRT1* was the best reference gene for retinal RT-qPCR (van Wijngaarden et al., 2007). A recent study aimed to identify the most stable reference genes for the normalization of target genes in the heart and liver of chickens exposed to heat stress and found that *RPL5,* and not *B-actin* or *HPRT1,* was the most stable reference gene for both heart and liver tissues (Gromboni et al., 2020).

Our results showed that *GAPDH*, followed by *RPL5*, was the best gene normalizer for retinal tissues, as they had the lowest and most consistent Ct values in both control and heat-treated chickens (Figures 3, 4). Therefore, *GAPDH* was chosen as the normalizer for RT-qPCR in the summer of 2020, and *RPL5* was chosen as the normalizer for RT-qPCR in the summer of 2021. Regardless of the reference gene normalizer used, we obtained a similar pattern of expression of HSP genes for the 21- and 35-day-old retinas in the summer of 2020 and 2021 (Figures 5–10), indicating that both *GAPDH* and *RPL5* can be used as reference gene normalizers for retinal tissues in heat stress experiments.

HSPs are a superfamily of stress proteins that promote cell survival and protect cells from thermal damage. Many studies correlate the overexpression of some HSP genes with protection against stress (Staib et al., 2007). Stress stimuli, such as hyperthermia, hypothermia, ischemia, hypoxia, depletion of ATP, free radicals, desiccation, steroid hormones, and ethanol, induce the expression of HSPs (Piri et al., 2016; Bellini et al., 2017). The cytoprotective roles of HSPs are related to their molecular chaperone functions (Piri et al., 2016; Murugesan et al., 2017). Molecular chaperones suppress protein aggregation and refold proteins into their native conformations. The well-studied molecular chaperones belong to the HSP family (Li et al., 2009; Murugesan et al., 2017).

HSP27 is a small heat shock protein that acts as a molecular chaperone by maintaining the denaturation of the protein in a folding-competent state. Moreover, it protects cells from oxidative stress and heat shock (Rogalla et al., 1999). HSP27 regulates apoptosis under stressful conditions and acts as an antioxidant when cells are exposed to oxidation and chemical stress (Vidyasagar et al., 2012). In the rat retina, HSP27 is upregulated under ischemic conditions and acts as a cytoprotective factor that prevents retinal damage (Whitlock et al., 2005). HSP27 suppression protected photoreceptor cells of the retina from apoptosis and restored retinal function in a light-induced rat retinal regeneration model (Chien et al., 2017). HSP27 was also upregulated in the retina of rats subjected to various models of retinal ganglion cell injury (Chidlow et al., 2014).

Considering HSP27 expression in relation to heat stress, previous studies have focused on cell lines exposed to chronic heat stress. In cardiac cells of hamsters subjected to hyperthermic treatment, HSP27 expression increased two-fold as compared to untreated cells (Ferns et al., 2006). Similarly, in cardiac cells of goats subjected to heat treatment, an increase in *HSP27* mRNA when compared to the control was noted (Parida et al., 2020). Furthermore, when neonatal rat primary myocardial cells were subjected to heat stress *in vitro, HSP27* mRNA levels increased after prolonged exposure to heat (Tang et al., 2013)*.*

To the best of our knowledge, this study is the first to report the expression of *HSP27* in the retina after exposure to chronic heat stress. Our data from the summers of 2020 and 2021 in Kuwait showed that *HSP27* was upregulated in 14-, 21-, and 35-days old retinas of the heat-treated group when compared to that of the control group. The most upregulation was seen at 14-days with almost a two-fold change; however, at 28-days, *HSP27* expression was downregulated (Figures 5, 6). This result might indicate similar roles of HSP27 in protecting the retina from damage and apoptosis upon exposure to chronic heat stress. However, the reason underlying the decrease in the expression of *HSP27* at 28-days of age is unclear, especially given that *HSP27* expression was upregulated at 21-days and significantly upregulated at 35-days (*p* = 0.024).

It has been reported that the expression of some HSP genes can fluctuate under certain heat treatments. For example, αB-crystallin, a small HSP gene closely related to HSP27, is downregulated in the heart of rats after 20 min of heat exposure, followed by upregulation at 40 min, and then downregulation again at 60 min (Tang et al., 2014). Therefore, HSP27 expression may also fluctuate under heat-stress. Another reason may be owing to differences in the gene expression of HSPs in different regions of the same organ. For example, the expression levels of HSP70 and HSP90 were higher in the ileum than in the jejunum of chicken intestinal tissues (Varasteh et al., 2015). In another study, HSP27, HSP70, and HSP90 were differentially expressed in different areas of the central nervous system of scrapie-infected and control sheep (Serrano et al., 2011). Based on these studies, it can be suggested that at 28 days, the expression of HSP27 was downregulated in the retina but maybe it was upregulated in other eye regions, such as the cornea or optic nerve.

HSP60 is typically known as a mitochondrial chaperonin protein that works together with the co-chaperonin HSP10. This chaperonin complex is essential for newly imported mitochondrial protein folding. HSP60 and HSP10 have also been shown to localize in the extracellular space, cytosol, and nucleus (Meng et al., 2018; Caruso Bavisotto et al., 2020). Most studies on HSP60 in the retina have reported that its expression is associated with several retinal diseases, such as glaucoma (Tsai et al., 2019) and diabetic retinopathy (Mohammad and Kowluru, 2011). Herein, we observed an upregulation of *HSP60* in the retina of chickens in the heat-treated group at all developmental stages when compared to that in the control group (Figures 5, 8). The highest upregulation was observed at 14-days, with almost a two-fold change when compared to the control group and the other developmental stages.

To the best of our knowledge, this study is the first to report the expression of *HSP60* under chronic heat stress in the retina. Previous studies investigating acute heat stress in chickens reported an increase in HSP60 mRNA in different tissues; for example, HSP60 was upregulated in the hearts of heat-stressed chickens within a few hours of treatment (Yu et al., 2008; Cheng et al., 2016). Similar findings were observed in the duodenum, jejunum, and ileum of chickens (Siddiqui et al., 2020). These results indicate that an increased HSP60 level may be an important marker at the beginning of heat stress and acts as protective in adverse environments (Yu et al., 2008).

HSP70 is a well-studied molecular chaperone and is highly conserved in all organisms (Franklin et al., 2005; Li et al., 2009; Rauch and Gestwicki, 2014; Radons, 2016). Moreover, it is the most common gene to be studied based on its response to different stressors (Zhao et al., 2013). HSP70 has three structural domains, including an N-terminal domain (ATPase domain) that binds ATP molecules, a substrate-binding domain that binds unfolded proteins, and a C-terminal domain that acts as a lid for the cavity. HSP40, however, is a co-chaperone protein and contains a J-domain, which enables it to bind to and regulate the ATPase domain in HSP70, thereby activating it (Li et al., 2009). Moreover, HSP40 coordinates other co-chaperones in binding HSP70, such as the HSP70-interacting protein (Lackie et al., 2017).

Most retinal studies have focused on HSP70, but not HSP40. Earlier studies in hyperthermic rats found that HSP70 mRNA was induced in the photoreceptor layer of the retina (Tytell et al., 1994). Later studies found that a brief period of hyperthermia in cultured cells and in the whole animal effectively induced HSP70 expression in the retina and significantly decreased photoreceptor degeneration in animals exposed to bright light (Piri et al., 2016).

Our data showed that under chronic heat stress, *HSP70* was upregulated throughout all developmental stages, 14-, 21-, 28-, and 35-days compared to the control (Figures 5, 9). The highest upregulation was observed at the earliest age (*p* = 0.039). *HSP40* was upregulated at the early stages, 14- and 21-days, but was downregulated at 28- and 35-days (Figures 5, 7). Our results may suggest that HSP40 is needed in the retina as a co-chaperone to activate HSP70 during the early stages of development; however, as retinal cells mature (recover or regenerate) within 28- and 35-days, there is no need for HSP40 expression, and HSP70 can function as the main chaperone independently.

Previous studies have reported that the expression of some HSP genes fluctuates across the developmental stages. For example, when 36 members of HSP family were examined in normal and abnormal development of embryonic hindlimbs, the RT-qPCR results showed that HSP27 and HSP40 expression levels changed from embryonic days 12–18 (Yan et al., 2015). As our chicks were developing from days 14–35, it could be possible that HSP27 and HSP40 were also differentially expressed throughout the different developmental stages. This may explain the sudden downregulation of HSP27 and HSP40 expression at 28-days, followed by the upregulation of HSP27 at 35 days, while the expression of HSP40 remained downregulated (Figures 6, 7).

HSP90 is one of the most abundant cellular chaperones and is involved in many cellular processes, such as folding, stability, maturation, maintenance, and degradation of several proteins. Additionally, it is the most conserved HSP that is essential for protection against heat shock response under normal and stress conditions (Retzlaff et al., 2009; Kanamaru et al., 2014; Hoter et al., 2018). In primate retina, HSP90 mRNA and protein are constitutively expressed at high levels in all layers of the retina and play a role in homeostasis. The retinal ganglion cells highly express HSP90 (Bernstein et al., 2001; Kanamaru et al., 2014). Prolonged inhibition of HSP90 has been shown to cause photoreceptor cell death (Zhou et al., 2013; Kanamaru et al., 2014). HSP90 and its inhibitors have been shown to prevent retinal degeneration in models of retinitis pigmentosa and age-related macular degeneration; therefore, they have been used as potential therapeutic agents for retinal diseases (Aguilà and Cheetham, 2016).

Herein, we found that under chronic heat stress, similar to *HSP70*, *HSP90* expression in the retina was upregulated in the heat-treated groups at all developmental stages when compared to the same developmental stages in the control group (Figures 5, 10). Furthermore, similar to that for *HSP70*, the highest upregulation was seen at the earliest age, i.e., at 14-days (*p* = 0.036).

The similar pattern of expression of *HSP70* and *HSP90* observed in our study was not surprising as many studies have linked the chaperone function of these two proteins. For example, the C-terminal HSP70-interacting protein is a co-chaperone for both HSP70 and HSP90 (Lackie et al., 2017). Moreover, recent studies have found that under stressful conditions, heat shock factor-1 dissociates from HSP90 to induce HSP70 expression, which leads to the correct refolding of misfolded proteins and/or their clearance to alleviate cell stress (Chaudhury et al., 2021). Additionally, our results on *HSP70* and *HSP90* expression under chronic heat stress in the chicken retina are consistent with their reported expression under heat stress in other tissues, such as the heart, liver, kidney, blood, intestine, and muscle (Yu et al., 2008; Lei et al., 2009; Xie et al., 2014; Varasteh et al., 2015; Cedraz et al., 2017).

In chickens, approximately 90% of the retina is produced 1 week before hatching. Therefore, chickens have fully functional retinas after hatching (Fischer and Reh, 2000; Fischer, 2005). A zone of cells at the retinal margin of postnatal chicks, called the circumferential marginal zone (CMZ), which contributes to the postnatal growth of the retina, has been identified and characterized (Fischer and Reh, 2000; Miles and Tropepe, 2021). Previous studies have shown that the addition of new cells to the edge of the retina continues for at least 3 weeks after hatching, and after this time period, fewer cells are added to this region. These findings indicate that the proliferation of progenitor cells and the addition of new cells to the retina in chickens decrease with increasing age (Fischer and Reh, 2000; Fischer, 2005). Earlier studies have also shown that some HSP genes are essential for normal retinal development and functional maturation of retinal cells at the embryonic and adult stages (Kojima et al., 1996; Sakai et al., 2003; Lee et al., 2006; Zhao et al., 2006).

Consistent with the findings of previous studies, our results showed that the highest upregulation of HSP genes (*HSP27*, *HSP60*, and *HSP70*) was at the earliest developmental stages of the retina (at 14-days) when compared to other developmental stages (Figures 6, 8, 9 respectively). We hypothesize that this elevated expression may serve dual functions in the earliest developmental stages of the retina to not only protect existing cells from damage, but also to promote regeneration, development, and maturation of new retinal cells in the CMZ region.

In conclusion, the present study was a two-year study that provided reproducible results on the expression of HSP genes under chronic heat stress. To the best of our knowledge, this is the first study to report the expression levels of *HSP27*, *HSP40*, *HSP60*, *HSP70*, and *HSP90* in the retina under chronic heat stress. Our data was consistent with the previously reported expression levels of some of these HSPs in other tissues, but not in the retina. The consistent increase in the expression of HSPs in the retina suggests their role as biomarkers for chronic heat stress. It is expected that the induction of HSPs in the retina during chronic heat stress is required to protect retinal cells from damage and degeneration. This study focused only on mRNA expression. Protein analysis is needed to confirm the function of HSPs in the retina under heat stress. Furthermore, it would be interesting to conduct the same study under acute heat stress and compare the expression of HSPs under both chronic and acute heat stress.

## Data Availability

The original contributions presented in the study are included in the article/Supplementary Material, further inquiries can be directed to the corresponding author.
